# Transcriptome profiling reveals the spatial-temporal dynamics of gene expression essential for soybean seed development

**DOI:** 10.1186/s12864-021-07783-z

**Published:** 2021-06-16

**Authors:** Hengyou Zhang, Zhenbin Hu, Yuming Yang, Xiaoqian Liu, Haiyan Lv, Bao-Hua Song, Yong-qiang Charles An, Zhimin Li, Dan Zhang

**Affiliations:** 1 Collaborative Innovation Center of Henan Grain Crops, College of Agronomy, Henan Agricultural University, Zhengzhou, 450002 China; 2 The Innovative Academy of Seed Design, Key Laboratory of Soybean Molecular Design Breeding, Northeast Institute of Geography and Agroecology, Chinese Academy of Sciences, Harbin, 150081 China; 3 Department of Biology, Saint Louis University, St. Louis, MO USA; 4 Department of Biological Sciences, University of North Carolina at Charlotte, Charlotte, NC 28223 USA; 5 US Department of Agriculture, Agricultural Research Service, Midwest Area, Plant Genetics Research Unit at Donald Danforth Plant Science Center, St. Louis, MO 63132 USA

**Keywords:** Soybean, *Glycine max*, Seed development, Transcriptome, Spatial/temporal gene expression

## Abstract

**Background:**

Seeds are the economic basis of oilseed crops, especially soybeans, the most widely cultivated oilseed crop worldwide. Seed development is accompanied by a multitude of diverse cellular processes, and revealing the underlying regulatory activities is critical for seed improvement.

**Results:**

In this study, we profiled the transcriptomes of developing seeds at 20, 25, 30, and 40 days after flowering (DAF), as these stages represent critical time points of seed development from early to full development. We identified a set of highly abundant genes and highlighted the importance of these genes in supporting nutrient accumulation and transcriptional regulation for seed development. We identified 8925 differentially expressed genes (DEGs) that exhibited temporal expression patterns over the course and expression specificities in distinct tissues, including seeds and nonseed tissues (roots, stems, and leaves). Genes specific to nonseed tissues might have tissue-associated roles, with relatively low transcript abundance in developing seeds, suggesting their spatially supportive roles in seed development. Coexpression network analysis identified several underexplored genes in soybeans that bridge tissue-specific gene modules.

**Conclusions:**

Our study provides a global view of gene activities and biological processes critical for seed formation in soybeans and prioritizes a set of genes for further study. The results of this study help to elucidate the mechanism controlling seed development and storage reserves.

**Supplementary Information:**

The online version contains supplementary material available at 10.1186/s12864-021-07783-z.

## Background

Soybean [*Glycine max* (L.) Merr.] is the most widely cultivated oilseed crop worldwide, and soybean seeds are a major source of highly valuable protein and edible oil for human and animal consumption [1]. Soybean production (bushels per acre) has nearly doubled since 1987 [2] and accounts for approximately 68 and 27% of world plant meal and oil production, respectively (www.fas.usda.gov/data/oilseeds), reflecting the key role of soybean seeds in providing food for the global population. Furthermore, it is estimated that a 70% increase in soybean production is needed to meet the increasing demand for plant-based protein in the next decades [3, 4]. It is important to understand the mechanism of seed development with the goal of continuously increasing soybean yield production and maintaining its sustainable role as a major source of protein and oil worldwide.

Soybean seeds are composed of functionally distinct tissues, including the embryo, embryonic cotyledon, and seed coat. These tissues are successively differentiated and grown during the maturation phase [5]. The filial embryo from double fertilization eventually grows into cotyledons that serve as storage organs for food reserves, mainly oil and protein. The seed coat is maternal tissue surrounding the embryo and cotyledon to provide protection and nutrient delivery in the form of photoassemblies to support the developing embryo [5, 6]. Ontogenesis and development of these tissues are molecularly programmed and correlate with parallel changes in nutrient levels in seeds. Seed storage reserves, such as stored proteins and lipids, are synthesized and accumulate during seed filling mainly from early to later maturation stages [7]. Overall, seed development is a complex developmental process accompanied by a multitude of molecular activities simultaneously occurring in coordinating tissue development and storage reserve metabolism.

Thus far, several genes involved in seed nutrient accumulation have been identified, such as bZIP123 involved in lipid accumulation and GA20OX associated with seed weight and oil accumulation [8–11], as well as genes associated with seed development [12]. Conservation of several regulatory processes of seed development between soybeans and the model plant, *Arabidopsis,* has been revealed [7, 13], thereby enabling homolog-based cloning and functional validation of related genes in soybeans [14, 15]. However, the functions of many soybean genes in seed development and nutrient accumulation have been modified in palaeopolyploid soybeans. For example, *ABSCISIC ACID INSENSITIVE 3b* (*ABI3b*) functions like LEAFY COTYLEDON 2 (*LEC2*) [16], and WRINKLED1 (WRI1) is associated with plant architecture instead of its homologous role in affecting oil production in *Arabidopsis* [14]. In contrast, each of these major regulators associated with seed development, such as LEC1, ABI3, ABA-RESPONSIVE ELEMENT BINDING PROTEIN3 (AREB3), and BASIC LEUCINE ZIPPER67 (bZIP67), putatively regulate thousands of targets involved in the control of diverse developmental processes through mosaic combinations of regulators [17]. These studies suggest that soybeans have evolved a complex regulatory network during seed development that is, in part, different from that in *Arabidopsis,* in which the current knowledge of plant seed development has been mostly obtained [18, 19]. Hence, the regulatory mechanism of seed development in soybeans is largely unclear and needs to be comprehensively explored.

Sequencing-based transcriptome profiling (RNA-seq) has been demonstrated to be an effective approach in dissecting the regulatory mechanisms of complex traits [20] and has been applied to investigate the changes in gene expression during seed development in legumes and oilseed plants [5]. For example, analysis of transcriptomes in developing soybean seeds has identified hub genes from a set of regulatory genes putatively participating in oil and protein accumulation [21, 22]. In the present study, we profiled the transcriptomes of progressively developing seeds at 20, 25, 30, and 40 days after flowering (DAF) to extend our knowledge of the molecular mechanism that controls seed development and nutrient production. We uncovered a variety of gene activities during the dynamic processes of seed development and identified multiple gene sets with spatial and temporal expression during the period. The results provide a global view of gene activity essential to the developmental process in soybeans, providing insight into the regulatory mechanism of seed formation.

## Results and discussion

### Dramatic morphology and reserve changes during seed development

**Fig. 1:**
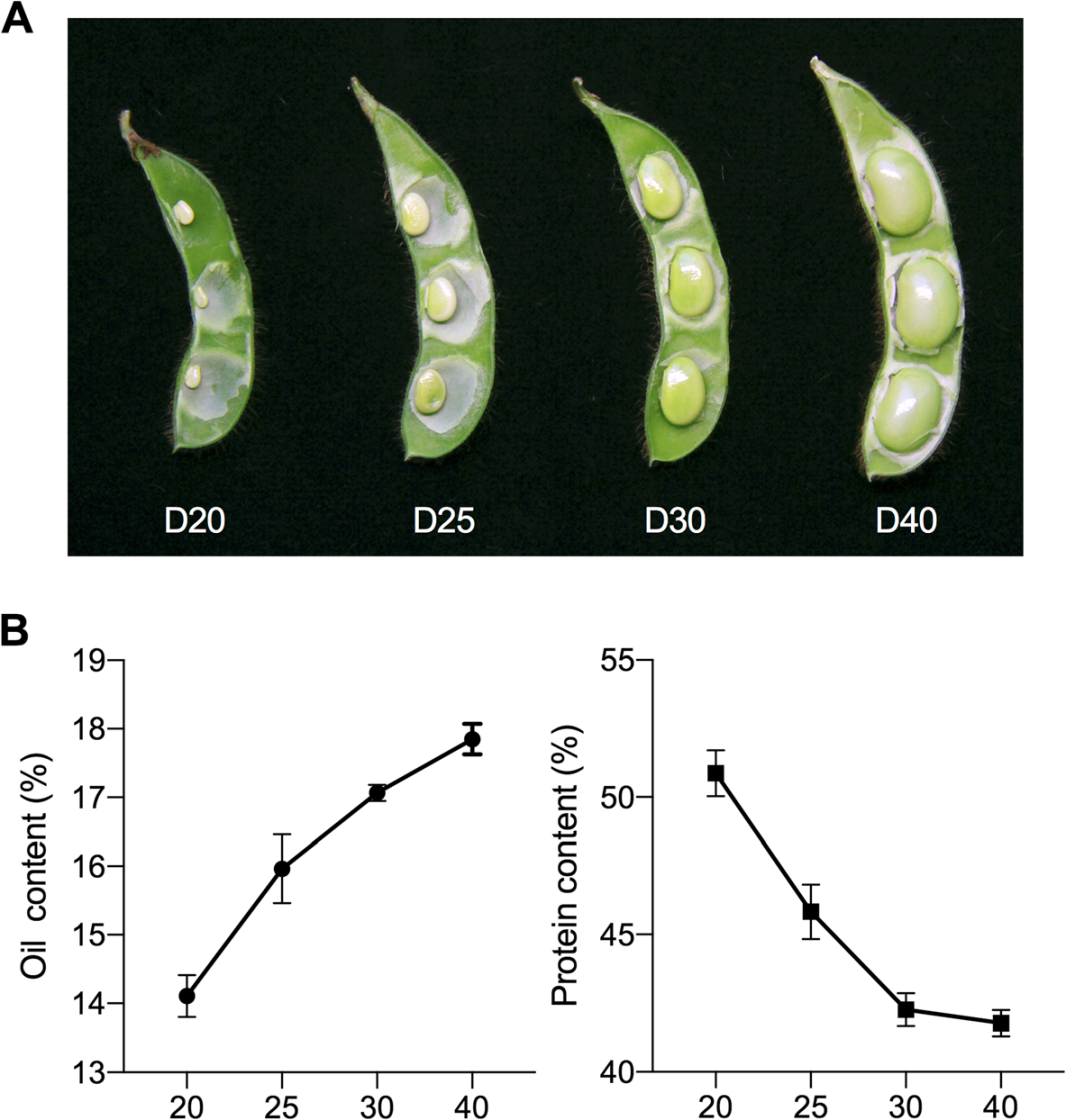
Morphology of developing seeds at 20, 25, 30, and 40 DAF (**A**) and changes in oil and protein during the selected stages (**B**)

Whole developing soybean seeds at four sequential time points (20, 25, 30, and 40 DAF) covering the major stages of seed development were sampled in triplicate (Fig. 1A). This period of seed development is coincident with seed growth stages from R5 (beginning seed) to R6 (full-size seed) of soybean development [23], which are morphologically comparable to early and mid-maturation stages as previously described [7]. This period also co-occurs with the rapid accumulation of lipids and storage proteins [20, 24]; thus, the seed size and seed weight increased significantly as it grew toward a fully developed seed (Fig. 1A). Two major storage reserves in soybean seeds, namely, protein and oil, were quantified during this period. As shown in Fig. 1B, the accumulation of stored oil was approximately 14.11% at D20 and continuously increased to 17.85% at D40 with the greatest increase of 1.85% occurring in the period from D20 to D25 relative to the later periods of D25-D30 by 1.11% and D30-D40 by 0.78%. In contrast, as the seeds matured, the stored protein content was 50.87% at D20, which decreased to 45.83% (5.04% decrease) at D25 and continuously declined to 42.26% at D30 and to 41.77% at D40. Thus, the oil content in the developing seeds increased more dramatically in the early period and less dramatically in the late period of seed filling, and vice versa for the protein decrease. The reduction in the relative percentage of protein content over the period is due to its negative correlation with the oil content in soybean seeds [25]. In contrast, the actual protein levels increased rapidly between 10 and 40 DAF [26]. Hence, this period of seed development presents rapid morphological changes (such as size and weight) and major metabolic changes (such as oil and protein), which should be governed by a variety of cellular processes/gene activities in seeds [17, 20].

### Transcriptomic abundance shifts during seed development

An in-depth understanding of biological processes associated with seed development and reserve accumulation in soybean seeds is important for improving seed quality and yield potential. Given the multiplicity of genes controlling seed development coupled with seed filling, time-course transcriptomic profiling of four-stage seeds that covered the representative period of seed development was performed with the aim of obtaining a global view of the regulatory mechanism underlying this complex development process. In total, ~ 313.4 million sequencing reads from all sampled seeds were generated, with an average of 26.1 million reads per sample (Table S1). On average, over 97.43% (25.4 million) of reads were kept after trimming the low-quality reads and used for read alignment. Uniquely mapped reads were used to quantify gene expression abundance as normalized by counts per million (cpm). After removing reads/transcripts < 5, we determined that, on average, 35,000 (62.5%) out of 56,044 gene models were expressed in developing seeds. We observed a gradient reduction (approximately 700 genes, 2% of the total) in the number of expressed genes over the course of seed development. The reduction could be, in part, related to the shutdown of many transcription processes as the seeds grow toward dormancy [27]. A previous study has reported a programmed increase in methylation levels during soybean seed development [7]. Thus, it would be interesting to determine whether the increased methylation level is related to the reduction of the seed transcriptome.

**Fig. 2:**
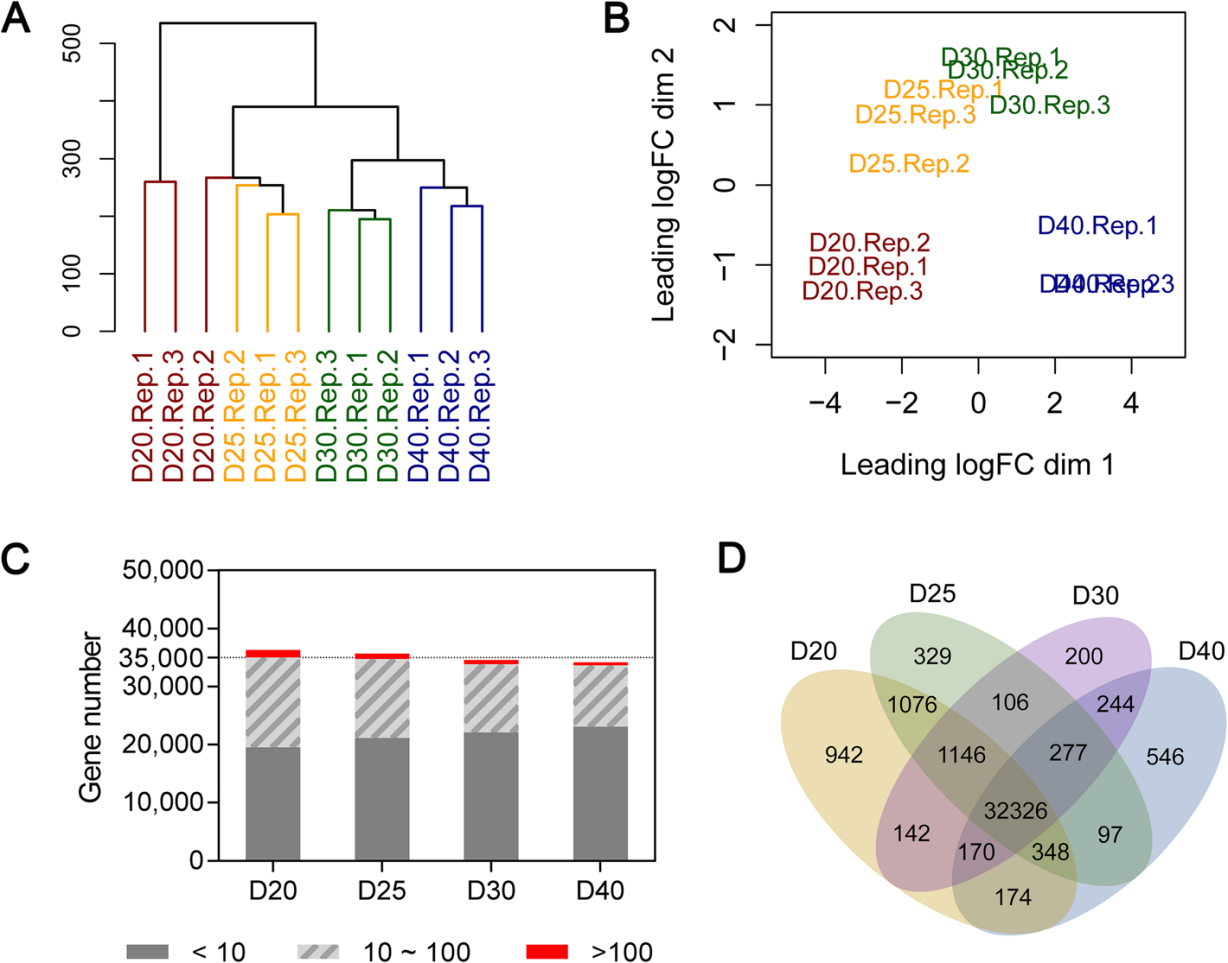
Gene expression profiles during seed filling. (**A**) Cluster dendrogram of gene expression profiles among biological replicates and four stages. (**B**) Multidimensional scaling plot of the collected samples across the four stages. (**C)** The number of genes expressed during each of the four stages. (**D**) A Venn diagram illustrating the number of expressed genes shared between or specific to the four different stages

Both clustering dendrogram analysis and multidimensional scale analysis indicated that gene expression levels among the replicated samples were generally highly related to those between time-point samples (Fig. 2A, B), indicating gene expression specificity to the time points. For the transcript abundance, approximately 61.07% of expressed genes were in the range of 0–10 cpm, 36.41% were in the range of 10–100 cpm, and only 2.53% of genes were highly expressed, at cpm ≥ 100 (Fig. 2C). As the seeds enlarged, the number of genes with an abundance ≥10 cpm generally decreased, and the number of genes with an abundance ≤10 cpm gradually increased. Among the expressed genes, 32,326 were expressed across all four stages of developing seeds. There were 942, 329, 200, and 546 genes specifically expressed at D20, D25, D30, and D40, respectively (Fig. 2D). These results suggest a temporal shift in transcript abundance for many highly expressed genes as seeds developed, which might reflect the foregoing morphological and/or metabolic changes during seed development.

A cluster dendrogram analysis showed that replicates from each condition were closely clustered as supported by a similar separation pattern in a multidimensional scaling (MDS) plot of log-FC values. We further performed *q*RT-PCR on 16 randomly selected DEGs (Table S4) for validation, and a high correlation (r = 0.87) between the RNA-seq and qRT-PCR results indicated the robustness of our RNA-seq results.

### Genes with high transcript abundance during seed development and filling

We next characterized the genes that were constitutively highly expressed across all four time points. In total, 443 genes with a mRNA abundance cpm ≥ 100 were identified [28] (Table S3). GO term enrichment analysis indicated that enriched biosynthetic processes (BPs) essential for nutrient accumulation were amino acid metabolism, carbohydrate metabolism, glycolysis, and hexose metabolism (Table S3). This gene set was also enriched for the molecular function (MF) term nutrient reservoir activity and cellular component (CC) terms related to lipid storage bodies, endoplasmic reticulum (ER), and ribosomes, sites where fatty acids are primarily synthesized and stored [29, 30]. When we relaxed the threshold to cpm ≥ 50, the number of newly defined highly expressed genes increased by threefold to 1386 genes (Table S3). Accordingly, the number of expressed genes in the majority of enriched terms was approximately doubled or tripled, such as hexose metabolic process (8 to 22), glycolysis (7 to 18), cellular nitrogen compound metabolic process (18 to 36), cellular amino acid biosynthetic process (15 to 32), and ribosomal proteins (47 to 132). It should be noted that no change in the number of genes with terms related to monolayer-surrounded lipid storage bodies (7), nutrient reservoir activity (11), and the sulfur amino acid metabolic process (6 to 7) was observed, suggesting the importance of maintaining high transcript abundances of these genes for seed filling. Additional enriched GO terms for the 1386 genes identified were as follows: protein localization, glutamine family amino acid biosynthetic process, intracellular transport, gene silencing by RNA, and vesicle-related components (COP-II-, COP-I, coated vesicles, transport vesicles, ER to Golgi transport vesicles, and membrane-bound vesicles).

A detailed investigation of the 443 genes (cpm ≥ 100) revealed several with demonstrated roles in the accumulation of storage reserves, such as oil, protein, and carbohydrates. Not surprisingly, some genes participating in the metabolism of protein and oil were identified. However, several of the highly abundant genes detected during seed filling have rarely been reported (Table S3), such as the oil accumulation-related genes, *FAD2* (467.83–2000.69) catalyzing oleic acid accumulation [31], OLEOSIN proteins (373.03–6190.57 cpm) encoding the oil body surrounding lipid storage bodies [30, 32], and metabolic genes for the biosynthesis of leucine, thiamin (vitamin B1), and ribosomal proteins. Genes encoding storage proteins, such as cupin family proteins, showed the highest transcript abundance, ranging from 34,000–100,000 cpm, in developing seeds followed by xylem bark cysteine peptidase 3 (*Glyma.08G116300* with 28,399.81 cpm), trypsin inhibitor 1 (222.79 ~ 18,559.51 cpm), and oleosin family proteins, which were highly abundant (373.03–6190.57 cpm). These proteins in soybeans appear to be redundant and serve as a surfactant for the oil body [33, 34]. A recent study on oleosin on chr20 [32] has indicated that it is likely regulated by LEC2 [15], by which oil production is enhanced through increasing oil body turnover. Investigation of the cis-regulatory sequences in the promoter region could be helpful in using this set of genes to achieve oil content improvement. In contrast, a set of highly abundant trypsin inhibitor genes (222.79–18,559.51 cpm) could be silenced by gene editing to inhibit their expression or mutate them to achieve better seed quality because trypsin inhibitors have an antinutritional effect [35].

More attention should be given to nutrient transporters that mediate nutrient flux into the developing embryo, such as the *SWEET* sugar transporters (274.98–943.19 cpm), proton pump interactor 1 and *SUCROSE SYNTHASEs* (121.5–1,186.21 cpm) controlling starch synthesis. Recent studies have highlighted the essential role of SWEET proteins in transporting sugar from maternal to embryonic tissues to support seed development and oil accumulation [12, 36, 37], indicating the agriculturally critical role of causing high oil accumulation in cultivated soybean seeds relative to the low oil content in wild soybeans [11]. High abundances of these transporters, together with related studies, suggest that the rate of sugar transport and sugar accumulation is important for nutrient reserve accumulation in seeds. Further investigation of downstream affected steps/components may help in identifying the major route of carbon flow for oil accumulation or carbon partitioning between oil and proteins in soybeans and, perhaps, other plant species, such as maize and rice [38, 39]. Several *LOX1* genes (541.27–16,226.05 cpm) had high transcript abundances in the present study, but homologous proteins have not been detected during seed filling in wheat and maize endosperms [40, 41], suggesting a lineage-specific role of LOX proteins in soybean seeds, possibly related to seed storage proteins and lipids, such as unsaturated fatty acids [42, 43], or special tastes and odors [44].

Transcription factors, such as ABA INSENSITIVE 3 (ABI3; *Glyma.08G357600*; *Glyma.18G176100*) and its interacting regulator, bZIP67, were identified in high abundance. The high abundance of both regulators may be due to the increasing need for their involvement in cell growth and the accumulation of storage reserves as seeds enlarge. Indeed, both regulators have common functions and are essential for initiating gene expression changes for biological processes critical for seed development, such as gibberellic acid signaling, photosynthesis, and maturation-related processes [16, 17, 45, 46]. LEC2s (cpm = 0) were undetectable in this study, possibly because they are highly specific to certain seed compartments and barely detected in whole seeds [15]. In addition, well-documented genes homologous to LEC1, which is a central regulator of seed development [17], were not in high abundance but showed decreased expression over time. Other high-abundance regulators with demonstrated roles in *Arabidopsis* that are less explored in soybean seeds were also identified, such as PLASTID TRANSCRIPTIONALLY ACTIVE 9 (PTAC9), TANDEM CCCH ZINC FINGER PROTEIN 4 (TCZF4) and circadian rhythm regulators, including TIME FOR COFFEE (TIC), LATE ELONGATED HYPOCOTYL 1 (LHY1), and CONSTANS-LIKE 4 (COL4). Determination of the functions of the regulators and the cognate cis-regulatory elements may facilitate the identification of seed regulatory networks that have contributing roles in the regulation of genes temporally and spatially in developing seeds [17, 18, 47]. All highly expressed genes and the functional annotations are listed in Table S2.

### Genes are temporally regulated during seed development

**Fig. 3:**
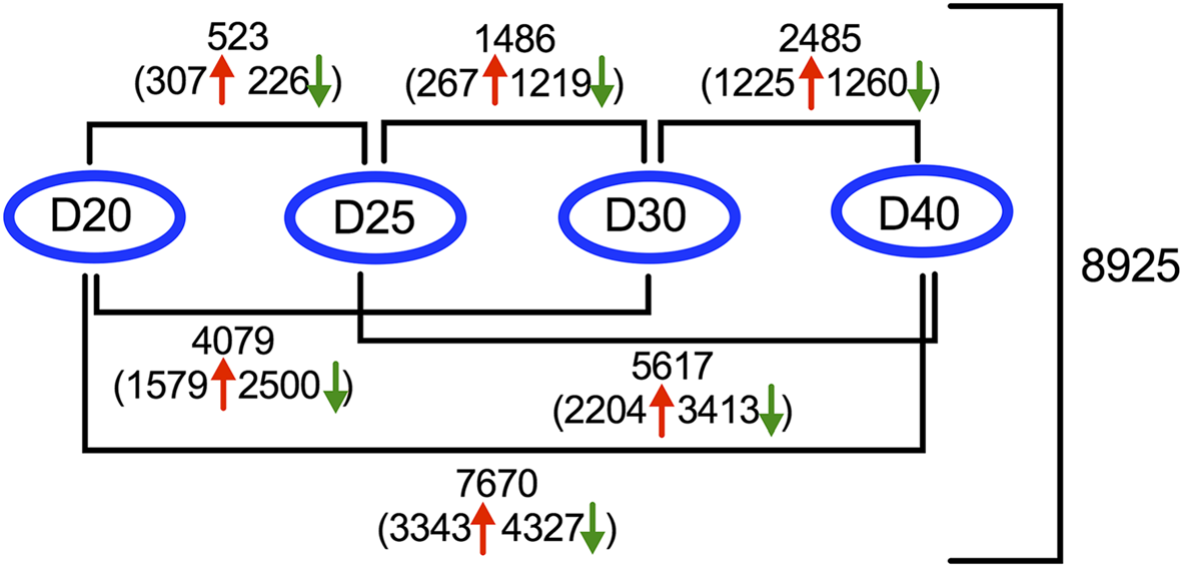
Number of DEGs estimated by pairwise comparison of the four stages

To comprehensively identify genes involved in developmental processes during seed filling, we conducted a pairwise comparison of transcriptomic profiles among the different developmental stages of seeds. We observed a gradient increase in the number of DEGs between the two neighboring time points as the seeds developed, with 523 in D25 vs. D20, 1486 in D30 vs. D25, and 2485 in D40 vs. D30 (Fig. 3). When comparisons were made across nonadjacent time points (over 10-day intervals), including 4079 in D30 vs. D20, 5617 in D40 vs. D25, and 7670 in D40 vs. D20, the numbers of DEGs increased. In total, the pairwise comparison identified 8925 nonredundant DEGs that are required to program soybean seed development from early to full seed development (Table S2).

**Fig. 4:**
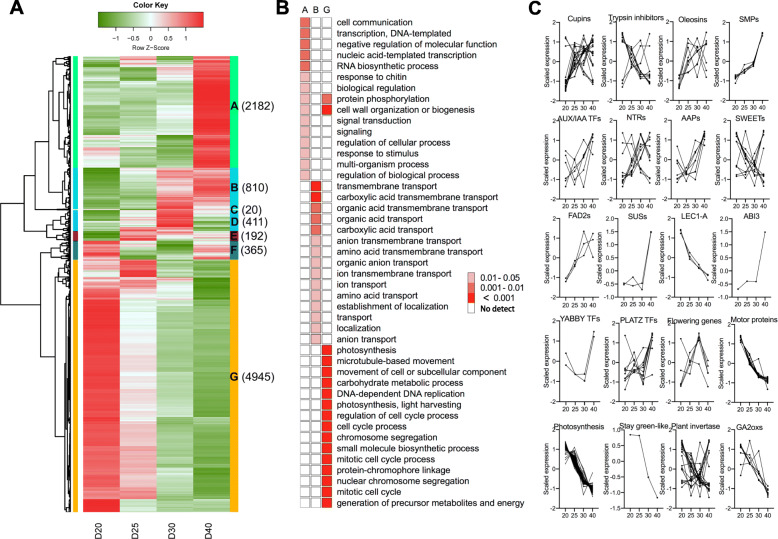
Temporal expression patterns of all DEGs. (**A**) Clustering of gene expression patterns across four time points during seed development. (**B**) GO terms enriched for the large clusters A, B, and G. (**C**) Expression patterns of selected genes

Clustering analysis was performed for all 8925 DEGs based on expression and is shown in a heat map (Fig. 4). The analysis classified the DEGs into seven groups (A to G) exhibiting different gene expression patterns. The difference in the temporal expression patterns suggested stage-specific roles of these grouped DEGs throughout seed development. Groups A and G represented the two largest clusters (2182 and 4945 DEGs, respectively) while exhibiting contrasting expression patterns. The DEGs in cluster A displayed the predominant and highest expression levels at D40, and those in group G showed a continuous decrease in gene expression as seeds matured. Enrichment analysis for cluster A genes showed that D40-specific genes were enriched for cell communication (GO:0007154) and transcription (GO:0006351), and the enriched biological processes occurred in the cell periphery and plasma membrane (Fig. 4B). This stage represented the later stage of reserve production and storage as well as the active period of the shift to maturation in which multiple programming might be initiated, such as cessation of organ differentiation, cessation of cell division, and preparation for seed desiccation [48]. A representative gene with specifically high expression at D40 is ABI3 (Fig. 4C) [16], which is an essential regulator in abscisic acid (ABA) signaling primarily participating in seed maturation and dormancy in *Arabidopsis* [49]. The high abundance of AB13 during D20-D30 and dramatic increase toward D40 suggested its critical role during seed development, especially in the later stage of maturation-related processes. ABI3 has recently been demonstrated to be a key transcription factor modulating the transition from morphogenesis to maturation by combinational interaction with other seed transcription factors [17]. Further study of the coexpressed genes or binding sites [17, 21] would provide an opportunity to understand regulation in soybean seeds. Identification of the key seed development regulators (such as ABI3) indicated the robustness of our analysis and suggested that other seed development-associated genes identified in our study deserve further attention. For example, a similar expression pattern was also observed for two sucrose synthases (SUSs) involved in cellulose and starch biosynthesis in seeds [50].

Genes classified in cluster B (810) were highly expressed at D40 and had a similar expression pattern to that in cluster A, except for those genes that were upregulated beginning at D30. Enriched GO terms for cluster B were transmembrane transport (GO:0055085) and nutrient reservoir activity (GO:0045735), suggesting that biological processes associated with nutrient transport, such as sugar transporters, amino acid transporters, and nutrient accumulation, were active beginning at D30 and maintained at D40. The developmental period of D30-D40 is approximately coincident with the middle-maturation stage, which represents the major period of accumulation for storage, such as that of protein and lipids [7]. Expression pattern analysis showed that despite the varying expression patterns observed, genes involved in the aforementioned biological processes showed obvious upregulation during seed development and filling (Fig. 4C), such as cupin proteins, oleosins, amino acid permeases (APPs), FAD2, nitrate transporters, and seed maturation proteins (SMPs). Such stark upregulation in expression undoubtedly reflects the critical roles of the related biological processes in storage reserve accumulation. Transcription factors involved in seed development were also upregulated in cluster B, such as the AUS/IAA transcription factor, YABBY, and uncharacterized PLATZ. Interestingly, the genes related to flowering (TFLs, Tof12, and PHYA) showed an upregulation/downregulation shift during the course, suggesting expanded roles during seed development, but this idea has yet to be explored. This finding is partially supported by a recent study demonstrating that *Arabidopsis TFL1* not only functions as a shoot identity gene but also serves as a signaling molecule essential in determining seed size, and the *tfl1–20* loss-of-function mutant has been shown to produce larger developing seeds than wild type through regulation of endosperm cellularization [51]. The expression patterns for these DEGs during seed development are illustrated in Fig. 4C.

In contrast, the top GO terms enriched for the genes in cluster G are mainly related to photosynthesis (GO:0015979) and microtubule-based processes (GO:0007017). Downregulation of these gene sets mainly reflect the reduction of the photosynthetic process as seeds developed, which as evidenced by downregulation of the bulk of genes encoding varying processes of photosynthesis, such as chlorophyll A-B binding family proteins, high-chlorophyll fluorescent proteins, and light-harvesting complex photosystem II (Table S1). Other family genes exhibiting downregulated expression patterns were also identified, such as GA2OXs, motor proteins, LACS9s, stay green-like genes, and LEC1-like genes (Table S1). The decrease in expression of these genes with diverse roles associated with phytohormones (such as GA) [52], chlorophyll breakdown during senescence [53], and seed developmental processes [47] might correspond to multiple processes of gradual cessation of cell division or commencement of maturation as seeds develop and mature. Clusters C-F comprised relatively fewer DEGs that displayed relatively high expression levels at two of the four selected time points, and no GO term was significantly enriched.

### DEGs exhibit spatial expression patterns in seed compartments and nonseed tissues

We next examined the spatial expression profiles of all DEGs in different developmental tissues using the Harada-Goldberg soybean RNA-seq dataset (http://seedgenenetwork.net). This dataset contains 10 tissues of Williams 82, which is the same accession used in our study, including 6 reproductive tissues [floral buds (FB) and five stages of developing seeds, including whole-mount globular (GLOB), heart (HRT), cotyledon (COT), early-maturation (EM), and dry seed (DS)] and four vegetative tissues (leaf (LF), root (RT), stem (STM), and seedling (SDL)). Although this study was performed independently, it was complementary to ours, and integration of our results with spatial analysis using multiple tissues may provide additional insight into the mechanism.

**Fig. 5:**
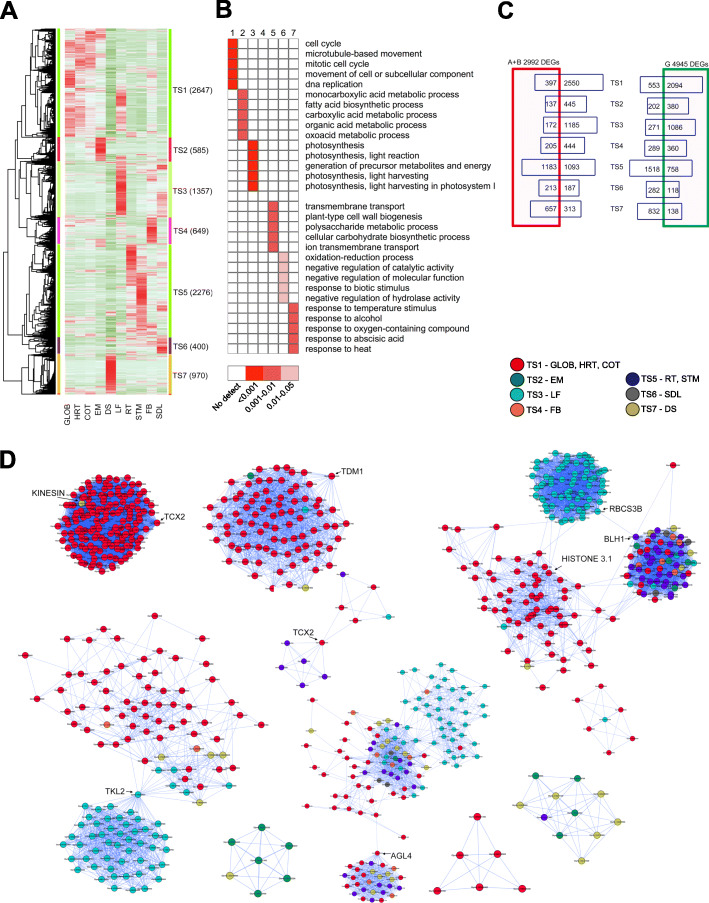
Tissue-specific expression patterns of all DEGs. (**A**) Clustering of the DEGs in different tissues, including globular (GLOB)-, heart (HRT)-, cotyledon (COT)-, and early maturation (EM)-stage seeds as well as leaf (LF), root (RT), stem (STM), floral bud (FB) and seedling (SDL). (**B**) Top five GO terms (biological process) enriched for the clustered genes. (**C**) Venn diagrams for TS clustered genes with clustered genes identified in A and G in Fig. 4. (**D**) Coexpression modules reconstructed by DEGs with the node color assigned by tissue

The DEGs exhibited clear tissue-specific (TS) patterns, as indicated by the higher expression in certain tissues compared to others. This analysis grouped the DEGs into seven large (TS1-TS7) groups with each containing over 400 genes, and the remaining three groups (not shown) comprised less than 30 genes (Table S2). The seven large groups, which were further investigated, are shown in Fig. 5A. Enrichment analysis for the seven TS groups revealed that no common top five enriched GO terms, indicating possible certain roles in the highly expressed tissues. Interestingly, genes that were highly expressed in developing seeds were divided into three groups: TS1 grouped genes highly expressed in three early-stage developing seeds, such as GLOB, HRT, and COT; TS2 grouped genes highly expressed in the EM-stage seeds; and TS3 grouped genes highly expressed in the later-stage seeds and DS-stage seeds. Approximately 2647 (29.7%) of the DEGs were clustered into TS1, consisting of genes primarily expressed in GLOB-, HRT-, and COT-stage seeds, and these genes showed continuous expression with either upregulation or downregulation. The majority (79.1%, 2094 of 2647) of TS1 was classified into the G group (downregulation group) (Fig. 5A, C), exhibiting downregulation as seeds grew. Genes (585, 6.6% of total) in TS2 were predominantly expressed in the EM-stage seeds. In total, 970 (10.9%) DEGs were exclusively expressed in DS-state seeds in TS7. This spatial difference in expression among the different stages of seeds suggested the possible different roles of the two gene sets in seeds. Indeed, TS1 genes are mainly involved in processes related to the cell cycle and DNA replication, which might account for early seed compartment morphogenesis and growth (Fig. 5B), and TS2 (EM) was more specialized in metabolic processes, such as fatty acid biosynthesis and metabolism of carboxylic acids and organic acids. In contrast, genes highly expressed in DS-stage seeds participate in the response to environmental stimuli, such as temperature, heat, alcohol, and plant hormones, which regulate plant growth, such as abscisic acid, and over half (67.7%) of this gene set was upregulated (Fig. 5C). For example, LEA14 (LATE EMBRYOGENESIS ABUNDANT 14) from TS7 is involved in wounding and light stress as well as possible protection against desiccation [54, 55]. Thus, the DEGs highly expressed in DS-stage seeds might be partially related to preparing transcripts for dormancy and, possibly, seed germination under favorable conditions.

Recent studies have indicated that the expression of many of these seed-expressed genes is highly spatially specific in seeds, and this specificity represents functional relevance. For example, seed coat-specific *SWEET39* is involved in sugar delivery from maternal plants to developing embryos [56] and endosperm-specific *TFL1* in endosperm cellularization, which affects seed size [51]. In addition, endosperm-exclusive SWEET15 participates in seed development by regulating sugar delivery into developing embryos [12]. These studies suggest that many of the genes highly expressed in seed compartments, as identified here (Table S2), can be prioritized for detailed investigations to gain a more comprehensive understanding, including YABBA, GA3OX1, NF-YB6, NF-YA9, FAD8, KAS I/III, SWEETs, OLEOSINs, AATs (amino acid transporters), and uncharacterized LEC1-LIKE (Glyma.07G268100, Glyma.17G005600), which serves as a key regulator of fatty acid biosynthesis in *Arabidopsis* [57].

In total, 1357 (15.2%) DEGs clustered into TS3 and were mainly expressed in leaves; they are involved in many aspects of photosynthesis with over 80% of the DEGs downregulated as seeds matured (Fig. 5C). These results indicated that this gene set maintain photosynthetic roles and is also associated with seed developmental processes. It is likely that the expression of many of these photosynthesis-related genes in seeds might be influenced by mosaic interactions of different transcription factors, such as LEC1, ABI3, bZIP67, and AREB3 [17, 47]. Genes that were highly expressed in the RT and STM shared many DEGs in common and clustered in TS5, which agreed with the tissue functionality of both being involved in nutrient translocation [58]. These genes are mainly involved in carbohydrate and sugar metabolism as well as nutrient and ion transport to meet the large demand for nutrient uptake and transport (Fig. 5B). TS6 was the smallest group (400), with genes highly expressed in SDLs and participating in processes such as oxidation-reduction. These results indicated that genes that have relatively high expression in nonseed tissues, such as LF, FB, and RT, might also maintain tissue-represented functions in developing seeds (Fig. 5B); however, their expression is relatively low in developing seeds, suggesting their indispensable roles in seed development. For example, AAP2 from TS6 (stem and leaf) affects the levels of nitrogen and carbon assimilation as well as seed yield by altering xylem-phloem transfer in amino acid transfer [59], and FRD3 (MATE efflux family protein) from TS6 functions in the root xylem for efficient iron uptake from the xylem into leaf cells [60]. Therefore, the identification of these genes with nonseed tissue specificities in developing seeds was reflective of the necessity of their basic functionalities in maintaining the needed processes to support seed development. Further investigation of these genes may provide deep insight into their supportive roles during seed development.

As indicated in this and other studies [17, 61], multiple layers of gene activities are involved in seed development, and the expression of the genes is also subjected to environmental effects. Despite promising genes identified from integrative analysis, it is ideal to compare the transcriptomes in different seed compartments and nonseed tissues at each of the time points in the same environment, which would offer a more comprehensive understanding of gene activities in developing seeds over the period.

### Coexpression network-inferred seed regulatory network

We next extracted the coexpression connections of all DEGs from a global SoyNet database (http://www.inetbio.org/soynet/) constructed with transcriptomes from different seed compartments at different developmental stages, along with other tissues [47, 62]. Only a small fraction of the DEGs could be reconstructed into modules. In total, 134 modules were identified with the numbers of nodes (genes) ranging from 2 to 151. After color assignment based on the aforementioned TS groups, 11 modules consisting of relatively tissue-specific patterns were identified (Fig. 5D). It is interesting that many genes from TS1 (GLOB, HRT, and COT) and TS3 (LF) were clustered together as individual TS-specific modules compared to the other TS groups. Two modules specific for TS1 were identified. TCX2 (TESMIN-LIKE CXC2) controls stem cell division by regulating stem cell type-specific networks [63]. TCX2 slightly deviated from the main module, suggesting its role in orchestrating the connected genes of this module for coordinated division of different cell types for seed development, which is in agreement with the function of GLOB/HRT/COT, where ontogenesis of different seed compartments occurs during this stage (http://seedgenenetwork.net/). Similarly, TDM1 plays an important role in meiosis termination [64], and it was strongly associated with genes in the TS1-dominated modules. We determined that uncharacterized TKL2 (TRANSKETOLASE 2) might be a key connector bridging two modules comprising LF and GLOB/HRT/COT genes. It is likely that the link between RBCS3B (RUBISCO SMALL SUBUNIT 3B) and BLH1 (EMBRYO SAC DEVELOPMENT ARREST 29) connected the two individual modules. These results suggest a functional relevance of these modules in seed development, and these genes that are underexplored in soybeans deserve more attention.

## Conclusions

In conclusion, we profiled the transcriptomes of developing seeds at four representative stages and provided a global transcriptomic view of the developmental process of storage reserve accumulation and seed development. The results highlight a set of highly abundant genes (storage genes, transporters, and transcription factors) that are important for soybean development and reveal that seed formation involves a variety of genes with tissue-associated roles to facilitate energy source transport, cell division, enlargement, and synthesis of highly abundant storage proteins and oil. Our study provides a global view of how a large number of genes are coordinated during seed development and may provide a framework for further investigations. Certainly, the role that these region-specific and period-specific genes and processes play in seed formation remains to be determined.

## Methods

### Plant growth and sample collection

The Williams 82 soybean variety was used in this study. Seeds were planted at the Maozhuang Experimental Station of Henan Agricultural University in 2018 in a three-row plot with row lengths of 200 cm and row spacing of 50 cm. A randomized block design was used for the field planting design. A flower on each node was tagged to indicate the flowering time. Developing seeds at 20 DAF (at the R5 stage) and at 25, 30, and 40 DAF (at the R6 stage) were collected [23], flash-frozen with liquid nitrogen, and stored in a − 80 °C freezer for RNA extraction. For RNA extraction, two to three developing seeds per plant were sampled and pooled as one biological replicate, and three biological replicates were used per time point. For protein and oil quantification, developing seeds from five plants (3–5 seeds per plant) were pooled as one biological replication; three biological replications were used. The crude extract and protein were quantified using the micro-Kjeldahl method and Soxhlet extractor, respectively, as previously described [65, 66].

### RNA isolation, library construction, and transcriptome sequencing

Total RNA was isolated using TRIzol reagent (Invitrogen, CA, USA) following the manufacturer’s protocol. Prior to library construction, total RNA was treated with RNase-free DNase I (New England Biolabs, Ipswich, MA, USA) to remove any contamination of genomic DNA. Library construction for RNA-seq was performed following a previously described protocol [67]. The quantified library was sequenced on the Illumina HiSeq 2500 sequencing platform (Illumina Inc., San Diego, CA, USA) at Biomarker Technologies (Beijing, China) and produced 200-bp paired-end reads.

### Data processing and differential expression

Raw sequencing data quality was evaluated with FastQC v0.11.5 (http://www.bioinformatics.babraham.ac.uk/projects/fastqc/) followed by removal of low-quality reads (quality value < 20) using Trimmomatic v0.33 [68]. The trimmed reads were aligned to the Williams 82 soybean reference genome (Wm82.a2.v1) [69] with TopHat (v2.1.1) using the minimum intron size (−i) parameter 30 and the maximum intron size (−I) of 15,000. The number of reads mapped to annotated genes was counted using featureCounts [70]. Analysis to determine differentially expressed genes (DEGs) was performed using EdgeR [71], and genes with an *fdr* ≤ 0.01 and a fold change ≥1.5 were considered significantly differentially expressed between the two conditions. Enrichment analyses for GO terms and KEGG pathways were performed in Gene Ontology (http://geneontology.org/docs/go-enrichment-analysis/) and KOBAS 3.0 (http://kobas.cbi.pku.edu.cn/), respectively, as previously described [72].

### Quantitative real-time PCR

Gene expression was examined in the same tissues used for RNA-seq by qRT-PCR as previously described [32]. Briefly, total RNA was isolated from the tissues using the RNA Simple Total RNA Kit (TaKaRa, Japan), and 1 μg of RNA was treated with RNase-free DNase I (TaKaRa, Japan) before cDNA synthesis. First-strand cDNA was synthesized using the SuperScript III First-Strand Synthesis System (Invitrogen, USA) following the manufacturer’s instructions. Gene expression was determined using the Bio-Rad CFX96 Touch Real-Time PCR System (Bio-Rad, CA, USA). The PCR mixture contained 5 μL of cDNA, 0.5 μL of 10 μmol L^− 1^ gene-specific primers, and 10 μL of Real-Time PCR SYBR Mix. The PCR conditions were as follows: 94 °C for 3 min and 40 cycles at 94 °C for 15 s and 60 °C for 15 s. The soybean *TUBULIN* gene (GenBank: AY907703.1) was amplified as an internal reference, and a negative control reaction was performed using water instead of cDNA. Three biological replicates per sample were used, and each reaction was performed in triplicate.

### Coexpression network construction

DEGs were extracted from the coexpression network in SoyNet that was preconstructed using transcriptomes from diverse tissues and seed compartments [62]. The coexpressed genes were reconstructed into modules using Molecular Complex Detection (MCODE) [73] using the default parameters. The module network was visualized using Cytoscape [74].

## Supplementary Information

**Additional file 1.**

## Data Availability

All data generated or analyzed during this study are included in this article and its additional files. The datasets generated and/or analysed during the current study are available in the Sequence Read Archive of the National Center for Biotechnology Information, “SRR9613714 - SRR9613721, SRR9613704, SRR13810418 - SRR13810420” under the BioProject: PRJNA551573. The Williams 82 soybean reference genome sequence was downloaded from the Phytozome v12 (https://phytozome.jgi.doe.gov/pz/portal.html). Data that was used for co-expression construction was retrieved from SoyNet (https://www.inetbio.org/soynet/).

## Abbreviations

BP: Biosynthetic processes
CC: Cellular component
COT: Cotyledon
cpm: Counts per million
DAF: Day after flowering
DEGs: Differentially expressed genes
DS: Dry seed
EM: Early-maturation
ER: Endoplasmic reticulum
GLOB: Globular
GO: Gene Ontology
HRT: Heart
LF: Leaf
MDS: Multidimensional scaling
MF: Molecular function
MCODE: Molecular Complex Detection
RT: Root
SDL: Seedling
SMPs: Seed maturation proteins
STM: Stem
TS: Tissue-specific
